# Systematic procedures including non-invasive ventilation improve morbidity in sleeve gastrectomy

**DOI:** 10.1186/cc14349

**Published:** 2015-03-16

**Authors:** I Auriant, N Devos, S Rossi

**Affiliations:** 1 Clinique de l'Europe, Rouen, France

## Introduction

Postoperative morbidity after sleeve gastrectomy is decreasing, but remains significant. Bleeding and surgical fistules remain the leading causes of morbidity and mortality. In several studies in postoperative care of obese patients, non-invasive positive pressure ventilation (NPPV) reduced the risk of lower respiratory tract infection and pneumonia [1], thereby reducing in-hospital morbidity. The aim of study was to describe whether systematic use of NPPV improves morbidity in the postoperative care of sleeve gastrectomy.

## Methods

A 4-year before-after study was conducted in a 19-bed intermediate care unit of a private hospital. Before period: standard treatment - all patients received oxygen supplementation to achieve SaO_2_ above 90%. After period: standard treatment plus NPPV - all patients were submitted to a systematic postoperative protocol: NPPV was provided using an oxygen CIPAP system with 5 cmH_2_O. Statistical analysis: complication rates were compared using the chi-square test. *P *< 0.05 was considered statistically significant.

## Results

A total of 857 patients were included. Inclusion characteristics were similar in the two groups: Before group - noNPPV: 352 patients, 2010 to 2011. Age: 40.58 ± 10.94, BMI: 42.79 ± 5.51, sex ratio F/M: 0.81. After group - NPPV: 504 patients, 2012 to 2013. Age: 40.81 ± 11.24, BMI: 42.92 ± 5.09, sex ratio F/M: 0.77. There is a significant between-group difference in the complication rate: Before group - noNPPV: 10 surgical fistula (2.84%) and six postoperative bleeding (1.70%); After group - NPPV: seven surgical fistula (1.39%) and three postoperative bleeding (0.6%). The overall complication rate fell from 4.54% to 1.98%. The chi-square statistic = 4.58. The number of degrees of freedom is 1. The value returned from the chi-square statistic is *P <*5%.

## Conclusion

Systematic use of NPPV significantly improves morbidity in the postoperative care of sleeve gastrectomy.
